# White matter microstructure alterations in frontotemporal dementia: Phenotype‐associated signatures and single‐subject interpretation

**DOI:** 10.1002/brb3.2500

**Published:** 2022-01-24

**Authors:** Mary Clare McKenna, Marlene Tahedl, Aizuri Murad, Jasmin Lope, Orla Hardiman, Siobhan Hutchinson, Peter Bede

**Affiliations:** ^1^ Computational Neuroimaging Group Trinity College Dublin Dublin Ireland; ^2^ Department of Neurology St James's Hospital Dublin Ireland

**Keywords:** amyotrophic lateral sclerosis, biomarker, frontotemporal dementia, MRI, neuroimaging

## Abstract

**Background:**

Frontotemporal dementias (FTD) include a genetically heterogeneous group of conditions with distinctive molecular, radiological and clinical features. The majority of radiology studies in FTD compare FTD subgroups to healthy controls to describe phenotype‐ or genotype‐associated imaging signatures. While the characterization of group‐specific imaging traits is academically important, the priority of clinical imaging is the meaningful interpretation of individual datasets.

**Methods:**

To demonstrate the feasibility of single‐subject magnetic resonance imaging (MRI) interpretation, we have evaluated the white matter profile of 60 patients across the clinical spectrum of FTD. A *z*‐score‐based approach was implemented, where the diffusivity metrics of individual patients were appraised with reference to demographically matched healthy controls. Fifty white matter tracts were systematically evaluated in each subject with reference to normative data.

**Results:**

The *z*‐score‐based approach successfully detected white matter pathology in single subjects, and group‐level inferences were analogous to the outputs of standard track‐based spatial statistics.

**Conclusions:**

Our findings suggest that it is possible to meaningfully evaluate the diffusion profile of single FTD patients if large normative datasets are available. In contrast to the visual review of FLAIR and T2‐weighted images, computational imaging offers objective, quantitative insights into white matter integrity changes even at single‐subject level.

## Graphical abstract

While the characterization of group‐specific imaging traits is academically important, the priority of clinical imaging is the meaningful interpretation of individual datasets. In this study, a *z*‐score‐based approach was implemented, where the diffusivity metrics of individual patients were appraised with reference to demographically matched healthy controls.

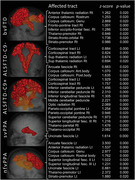

## 1 INTRODUCTION

White matter (WM) changes in frontotemporal dementia (FTD) have been extensively studied, and both clinical subtypes (Agosta et al., 2012; Borroni et al., 2007; Lam et al., 2014; Mahoney et al., 2014; Schwindt et al., 2013; Whitwell et al., 2010) and genotypes (Jiskoot et al., 2018, 2019; Rohrer et al., 2010) have been linked to relatively specific WM signatures. The most commonly utilized WM technique is diffusion tensor imaging (DTI), but a variety of non‐Gaussian techniques such as diffusion kurtosis imaging (DKI) and neurite orientation dispersion and density imaging (NODDI) have also been successfully utilized (Wen et al., 2019). WM alterations in FTD can already be detected in the presymptomatic phase of the disease, and WM alterations are relatively marked by the time the diagnosis can be established (Wen et al., 2019). WM changes can also be readily tracked longitudinally across multiple timepoints to appraise the rate of progression and patterns of anatomical propagation. A shortcoming of descriptive imaging studies in FTD is that often only group‐level inferences are presented, that is, shared patterns of WM disease burden in specific phenotypes or genotypes. The demands of clinical imaging differ significantly from the deliverables of academic radiology (Bede et al., 2018). The emphasis in the clinical setting is the accurate categorization of a suspected patient into a diagnostic subgroup, the evaluation of an asymptomatic mutation carrier with regard to presymptomatic disease burden, or the follow‐up of a specific patient with an established diagnosis to verify if further pathology has been accrued (Chipika et al., 2019; Chipika, Siah, et al., 2020). The gap between group‐level imaging and single‐subject imaging is considerable in terms of practical utility, methodological challenges and academic relevance (Verstraete et al., 2015). While patterns of gray matter atrophy can be assessed in a variety of ways, the interpretation of single‐subject WM profiles is particularly challenging. The visual inspection of FLAIR and T2‐wighted images offers limited information, and the visual review of DTI data only permits the appreciation of movement, susceptibility or eddy‐current related artefacts. In current clinical practice, the primary role of magnetic resonance imaging (MRI) is the exclusion of neoplastic, paraneoplastic, inflammatory and structural mimics rather than the confirmation of FTD‐associated changes. Existing frameworks for single‐subject categorization rely on various machine learning algorithms to classify single individuals into groups. A variety of supervised and unsupervised methods have been previously implemented across the spectrum of ALS‐FTD. Models such as support vector machines, decision trees, neural networks and discriminant function analyses have been applied to imaging datasets with varying accuracy (Bede et al., 2017; Bede, Murad, et al., 2021; Cajanus et al., 2018; Chagué et al., 2020; Davatzikos et al., 2008; Donnelly‐Kehoe et al., 2019; Egger et al., 2020; Feis et al., 2018, 2020; Feis, Bouts, de Vos, et al., 2019; Feis, Bouts, Panman, et al., 2019; Grollemund et al., 2019; Klöppel et al., 2015; Koikkalainen et al., 2016; Manera et al., 2021; Schuster et al., 2016, 2017; Tong et al., 2017; Torso et al., 2020; Vernooij et al., 2018; Young et al., 2018). A common application of these approaches is the categorization of patients into FTD versus AD diagnostic groups. (Bouts et al., 2018; Bron et al., 2017; Canu et al., 2017; Frings et al., 2014; Hu et al., 2020; Kim et al., 2019; Ma et al., 2020; Möller et al., 2016; Wang et al., 2016; Yu et al., 2021). A key barrier to the development of successful machine learning algorithms in neurodegenerative conditions is the scarcity of uniformly acquired training data, especially in low‐incidence phenotypes such as ALS‐FTD, PLS‐FTD, and post‐polio syndrome (Aho‐Ozhan et al., 2016; Finegan et al., 2019, 2021; Li Hi Shing, Lope, Chipika, et al., 2021; Li Hi Shing, Lope, McKenna, et al., 2021; Lule et al., 2010; McKenna, Corcia, et al., 2021; Pioro et al., 2020; Trojsi et al., 2015, 2019; Trojsi, Di Nardo, Siciliano, et al., 2020). Accordingly, the objective of this study is to pilot an alternative quantitative WM rating framework for single‐subject diffusion data interpretation based on tractwise *z*‐scoring of diffusivity metrics with reference to demographically matched controls.

## 2 METHODS

### 2.1 Participants

A total of 160 subjects were enrolled in this study. Sixty patients were included from across the clinical spectrum of FTD: seven patients with behavioral variant FTD (bvFTD, four males, mean age = 60.71 ± 3.30 years), nine patients with nonfluent variant primary progressive aphasia (nfvPPA, five males, mean age = 62.22 ± 3.03 years), three patients with semantic‐variant PPA (svPPA, two males, mean age = 61.67 ± 6.43 years), 21 patients with ALS‐FTD carrying hexanucleotide repeat expansions in *C9orf72* (ALSFTD‐C9+, 13 males, mean age = 58.95 ± 9.95 years) and 20 ALS‐FTD patients who tested negative for *C9orf72* (ALSFTD‐C9NEG, 13 males, mean age = 60.65 ± 8.73 years). The imaging profiles of patients were interpreted based on radiological data from 100 healthy controls (HC, 50 males, mean age = 58.95 ± 9.95 years). Patients were diagnosed according to the Rascovsky and El Escorial criteria. The *z*‐scoring strategy implemented in this study relies on the rating of single subjects’ data with respect to a demographically matched control population. Accordingly, control selection for normative data generation was defined based on age to ensure age matching between each male/female patient and the corresponding male/female control group. Two‐sample *t*‐tests were performed to verify successful age matching. Sex matching was ensured by contrasting each male/female patient only to male/female controls. Given the available number of total controls, only one male and one female control group were defined, each of size *n* = 50. Relevant demographic data are presented in Table 1. Methods for ascertaining GGGGCC hexanucleotide repeat expansion in *C9orf72* by repeat‐primed PCR have been described previously (Chipika, Christidi, et al., 2020; Kenna et al., 2013), and expansions longer than 30 hexanucleotide repeats were considered pathological.

**TABLE 1:** Demographic data

	Male		Female	
Patient group	Mean age (SD) (years), sample size (*n*)	*t*‐Score from two‐sample *t*‐test (DOF), *p*‐value (HC vs. patients)	Mean age (SD) (years), sample size (*n*)	*t*‐Score from two‐sample *t*‐test (DOF), *p*‐value (HC vs. patients)
ALSFTD‐C9+	55.92 (8.11), *n* = 13	*t*(61) = 1.73, *p* = .09	58.50 (9.61), *n* = 8	*t*(56) = −0.42, *p* = 0.68
ALSFTD‐C9‐	62.00 (9.11), *n* = 13	*t*(61) = −0.35, *p* = .73	58.14 (7.98), *n* = 7	*t*(55) = −0.31, *p* = .76
bvFTD	59.25 (3.50), *n* = 4	*t*(52) = 0.35, *p* = .73	62.67 (2.98), *n* = 3	*t*(51) = −0.99, *p* = .33
nfvPPA	63.60 (2.97), *n* = 5	*t*(53) = −0.60, *p* = .55	60.50 (3.42), *n* = 4	*t*(52) = −0.71, *p* = .48
svPPA	58.00 (1.41), *n* = 2	*t*(50) = 0.43, *p* = .67	69.00 (0.00), *n* = 1	*t*(49) = −1.21, *p* = .23
HC	60.96 (9.68), *n* = 50		56.94 (9.91), *n* = 50	

*Note*: ALSFTDC9‐: ALS‐FTD patients without *C9orf72* hexanucleotide expansions, ALSFTDC9+: ALS‐FTD patients with *C9orf72* hexanucleotide expansions

Abbreviations: bvFTD, behavioral variant FTD; DOF, degrees of freedom; HC, healthy control; nfvPPA, nonfluent variant primary progressive aphasia; svPPA, semantic variant primary progressive aphasia.

### 2.2 Data acquisition

A spin‐echo echo planar imaging (SE‐EPI) pulse sequence with a 32‐direction Stejskal–Tanner diffusion encoding scheme was used to acquire DTI data on a 3 Tesla Philips Achieva MR platform. The key parameters included TR/TE = 7639/59 ms, *b*‐values = 0.1100 s/mm^2^, FOV = 245 × 245 × 150 mm, spatial resolution = 2.5 mm^3^, 60 axial slices with no interslice gaps, SENSE factor = 2.5, dynamic stabilization and spectral presaturation with inversion recovery (SPIR) fat suppression. For the visual assessment of co‐morbid WM pathology, FLAIR images were also reviewed of each participant. FLAIR data were acquired in the axial orientation using an inversion recovery turbo spin echo (IR‐TSE) sequence: FOV = 230 × 183 × 150 mm, spatial resolution = 0.65 × 0.87 × 4 mm, TR/TE = 11000/125 ms, TI = 2800 ms, 120° refocusing pulse, with flow compensation and motion smoothing and a saturation slab covering the neck region. T1‐weighted (T1w) images were acquired with a 3D inversion recovery prepared spoiled gradient recalled echo (IR‐SPGR) sequence with a field‐of‐view (FOV) of 256 × 256 × 160 mm, spatial resolution of 1 mm^3^, TR/TE = 8.5/3.9 ms, TI = 1060 ms, flip angle = 8°, SENSE factor = 1.5.

### 2.3 Diffusion‐weighted data processing

Diffusion‐weighted (DW) data were preprocessed within *MRtrix3*, including noise removal and removal of Gibb's Ringing Artifacts. The *topup‐eddy* algorithm was utilized for corrections for eddy‐induced distortions and subject movements as implemented in FSL. Bias correction was performed with the ANTs1.9 *N4* method. Diffusion tensors were fitted within *MRtrix3*, and maps of fractional anisotropy (FA) and radial diffusivity (RD) were generated. Anatomical images were preprocessed using FMRIB's FSL6.0's *fsl‐anat* algorithm, including brain‐extraction and biasfield‐corrections.

### 2.4 Tract segmentation

As the main objective of the study was the detection of WM microstructure integrity changes in individual patients, our analyses were restricted to regions of FA reductions and foci of increased RD as these diffusivity shifts indicate pathologic processes. Tract‐wise probabilities of presumed pathology in individual subjects were estimated based on reference normative data. First, each patient's and control's DW data were segmented into 50 WM tracts using a neural‐network‐based algorithm, *TractSeg*, which, as opposed to atlas‐based approaches, does not assume a common anatomy between subjects and relies on individual WM fiber bundles anatomy. Peaks of the spherical harmonic function were extracted at each voxel to inform *TractSeg*, which were calculated from fitting voxelwise constrained spherical deconvolution (CSD). CSD is an alternative to the tensor model to perform tractography, which has been shown to outperform the tensor model in regions of crossing fibers, among others. Response functions were estimated using the *dhollander* method as implemented in *MRtrix3* from which fiber orientation distribution function (fODF) could be calculated. Given that DW shells were acquired (*b* = 1000 and *b* = 0), a multishell approach could be implemented. Resulting fODFs were normalized according to (Raffelt et al., 2017); spherical harmonic peaks were retrieved from the normalized measures, which then served as input values into *TractSeg*.

### 2.5 *z*‐Score‐based tract integrity evaluation

The concept behind the *z*‐scored‐based strategy is the ascertainment of affected fiber bundles in individual patients. WM tracts were rated in individual patients with reference to age‐ or sex‐matched HCs. Only tracts exhibiting significant FA reductions and increased RD were considered ‘‘affected.’’ First, subject‐specific FA and RD maps were created for the segmented tracts by inputting each subjects’ individual FA/RD map into *TractSeg* and averaging the estimated values across each tract. Normative data from HCs were *z*‐scored, and patient data were normalized with respect to the relevant control group. Single patients’ tract profiles were then contrasted to normative data using nonparametric statistics. First, the number of HCs exhibiting lower FA and higher RD than the observed value in the patient was determined for each patient and each tract. This value was then divided by the number of HCs (i.e., 50 both for males and females) to obtain *p*‐values. Given that two tests were run (decreased FA/increased RD), tracts with *p* < .025 were considered significantly different.

Finally, group‐level statistics were also derived from the *z*‐score‐based strategy to aid cross‐validation against the standard approach. We tested which tracts were preferentially affected across the entire patient group. To quantify this probability, probability distributions were first created reflecting the number of false positives across the patient group (i.e., *p*‐values of < .025 provided a random event). This was modeled as a binomial process:

(1) $$X∼B\left(n,p\right),$$

where *X* is the random variable (a scalar), *n* is the number of correctly segmented tracts in the control distribution and *p* is the probability of assigning significance to a tract's *p*‐value (in our case 0.025). This process was repeated 100,000 times to provide a dense probability distribution. *p*‐Values were then derived for each tract by counting how many values in the null distribution exceeded the sum of significant observations across the patient group and dividing that count by the number of iterations. To match the threshold used in the validation arm of the study, the most affected tracts were identified using a relatively stringent alpha‐threshold of *p* < .01.

### 2.6 Cross‐validation by standard tract‐based statistics

To validate the *z*‐score‐based approach, the group‐level outputs were compared to those of an established analysis pipeline, FMRIB FSL's tract‐based spatial statistics (TBSS). The voxelwise diffusivity profile of the five FTD groups was contrasted to controls. In accordance with FSL's TBSS recommendations, processing included outliner removal, nonlinear registration to the FMRIB58FA template and application of that transformation to align all subjects’ FA/RD images to the MNI152 1 mm standard space. Voxelwise group‐comparisons were computed using FSL's *randomise* algorithm, a nonparametric permutation testing scheme, with 2D‐optimized threshold‐free cluster enhancement (TFCE) to control for the family‐wise error rate (FWER). To highlight the most pertinent WM changes, a stringent alpha‐threshold of *p*_FWER _< .01 was applied.

## 3 RESULTS

### 3.1 Demographics

Two‐sample *t*‐tests were run between each male/female patient group versus the male/female control groups to confirm age matching. No statistical difference was found between any of the patient and control groups suggesting appropriate age matching. Relevant descriptive and inferential statistics are provided in Table 1.

### 3.2 *z*‐Score‐based subject‐level inferences

**FIGURE 1:**
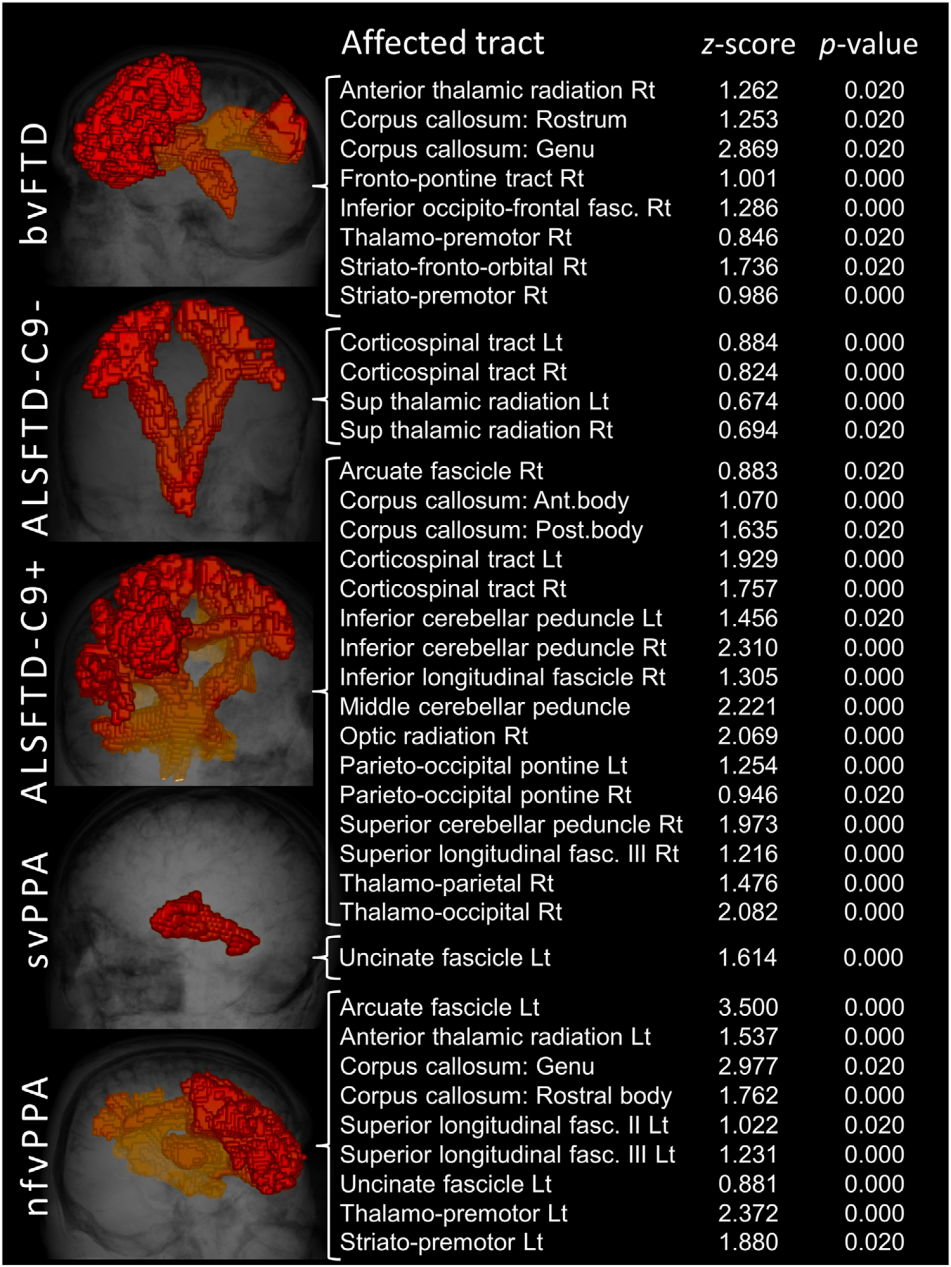
White matter alterations in individual subjects based on single diffusion tensor imaging (DTI) datasets and normative data. Illustrative outputs from single patients with behavioral variant FTD (bvFTD), ALS‐FTD patients without *C9orf72* hexanucleotide expansions (ALSFTD‐C9‐), ALS‐FTD patients with *C9orf72* hexanucleotide expansions (ALSFTD‐C9+), semantic variant primary progressive aphasia (svPPA) and nonfluent variant primary progressive aphasia (nfvPPA)

The *z*‐score‐based strategy has successfully captured relevant WM pathology in individual subjects in each of the 60 FTD patients. A dual‐output scheme was utilized. Affected WM tracts can be depicted in 3D, and a text file was also generated listing the affected tract with the relevant *z*‐ and *p*‐values. To showcase the potential utility of single‐subject WM profile interpretation, we provide representative individual examples from the five patient groups (Figure 1). As descried in Section 2, the *z*‐score‐based strategy also permits the description of group‐level findings. An overview of affected tracts at a group level is provided in Tables 2, 3 for each FTD cohort. More tracts were detected exhibiting increased RD than tracts with FA reductions, suggesting that RD may be more sensitive to capture relevant WM degeneration. Our approach detected left‐hemisphere dominant changes in language variant phenotypes (nfvPPA and svPPA) compared to the relatively symmetric pathology in bvFTD and ALS‐FTD groups. Relative sparing of posterior WM bundles was observed across the entire spectrum of subgroups.

**TABLE 2:** Affected white matter tracts in ALS‐FTD at group level

ALS‐FTD with GGGGCC hexanucleotide repeat expansions in *C9orf72* (C9orf72+)					
FA reductions		Increased RD		Increased RD (continued)	
Tract name	*p*_FWER_	Tract name	*p*_FWER_	Tract name	*p*_FWER_
Arcuate fascicle right	.0011	Arcuate fascicle left	.0001	Optic radiation right	<.0001
Corpus callosum: rostrum	.0096	Arcuate fascicle right	<.0001	Parieto‐occipital pontine left	<.0001
Corpus callosum: genu	.0009	Corpus callosum: rostrum	<.0001	Parieto‐occipital pontine left	.0008
Corpus callosum: anterior midbody	.0001	Corpus callosum: genu	.0010	Superior cerebellar peduncle right	.0008
Corpus callosum: posterior midbody	<.0001	Corpus callosum: rostral body	.0029	Superior longitudinal fascicle I left	<.0001
Corpus callosum: isthmus	.0011	Corpus callosum: anterior midbody	<.0001	Superior longitudinal fascicle I right	.0001
Corpus callosum: splenium	<.0001	Corpus callosum: posterior midbody	.0001	Superior longitudinal fascicle II left	.0012
Cingulum left	.0012	Corpus callosum: isthmus	.0011	Superior longitudinal fascicle II right	.0009
Cingulum right	.0013	Corpus callosum: splenium	.0010	Superior longitudinal fascicle III left	.0084
Corticospinal tract left	.0073	Cingulum left	<.0001	Superior longitudinal fascicle III right	.0001
Corticospinal tract right	<.0001	Cingulum right	.0013	Superior thalamic radiation left	<.0001
Fronto‐pontine tract right	.0082	Corticospinal tract left	<.0001	Superior thalamic radiation right	<.0001
Optic radiation left	.0008	Corticospinal tract right	<.0001	Uncinate fascicle left	<.0001
Parieto‐occipital pontine right	.0095	Fronto‐pontine tract left	.0085	Uncinate fascicle right	.0004
Superior longitudinal fascicle I left	.0001	Fronto‐pontine tract right	.0082	Thalamo‐parietal right	.0001
Superior longitudinal fascicle I right	<.0001	Inferior cerebellar peduncle left	.0001	Thalamo‐occipital left	<.0001
Superior longitudinal fascicle II left	.0010	Inferior cerebellar peduncle right	.0053	Thalamo‐occipital right	<.0001
Superior longitudinal fascicle II right	.0007	Inferior occipito‐frontal fascicle left	.0001	Striato‐fronto‐orbital right	<.0001
Superior longitudinal fascicle III right	<.0001	Inferior occipito‐frontal fascicle right	<.0001	**Right**	
Superior thalamic radiation right	.0005	Inferior longitudinal fascicle left	<.0001		
Thalamo‐occipital left	.0009	Inferior longitudinal fascicle right	<.0001		
Striato‐fronto‐orbital right	.0095	Optic radiation left	<.0001		

ALS‐FTD without GGGGCC hexanucleotide repeat expansions in *C9orf72* (C9orf72‐)					
FA reductions		Increased RD		Increased RD (continued)	
Tract name	*p*_FWER_	Tract name	*p*_FWER_	Tract name	*p*_FWER_
Arcuate fascicle left	.0097	Arcuate fascicle left	.0001	Parieto‐occipital pontine right	.0055
Corpus callosum: genu	.0007	Arcuate fascicle right	.0009	Superior cerebellar peduncle left	.0006
Corpus callosum: splenium	.0005	Corpus callosum: rostrum	<.0001	Superior cerebellar peduncle right	.0059
Cingulum left	.0009	Corpus callosum: genu	<.0001	Superior longitudinal fascicle I left	<.0001
Corticospinal tract right	.0002	Corpus callosum: rostral body	.0013	Superior longitudinal fascicle I right	.0007
Inferior occipito‐frontal fascicle right	.0068	Corpus callosum: isthmus	<.0001	Superior longitudinal fascicle II left	<.0001
Middle cerebellar peduncle	.0068	Corpus callosum: splenium	.0080	Superior longitudinal fascicle III left	.0060
Superior longitudinal fascicle III right	.0072	Cingulum left	.0001	Superior longitudinal fascicle III right	.0067
		Cingulum right	.0070	Superior thalamic radiation left	<.0001
		Corticospinal tract left	<.0001	Superior thalamic radiation right	.0047
		Corticospinal tract right	.0002	Uncinate fascicle left	<.0001
		Fronto‐pontine tract right	.0003	Uncinate fascicle right	<.0001
		Inferior cerebellar peduncle left	.0071	Thalamo‐parietal left	.0064
		Inferior cerebellar peduncle right	.0075	Thalamo‐occipital right	.0004
		Inferior occipito‐frontal fascicle right	.0005	Striato‐fronto‐orbital left	.0069
		Middle cerebellar peduncle	.0067	Striato‐premotor left	.0060
		Optic radiation right	.0004		

Abbreviations: FA, fractional anisotropy; RD, radial diffusivity.

**TABLE 3:** Affected white matter tracts in behavioral variant FTD (bvFTD), nonfluent variant primary progressive aphasia (nfvPPA) and semantic variant primary progressive aphasia (svPPA) at group level

Decreased FA		Increased RD	
Tract name	*p*_FWER_	Tract name	*p*_FWER_
bvFTD			
Corpus callosum: genu	<.0001	Arcuate fascicle left	.0004
		Corpus callosum: rostrum	<.0001
		Corpus callosum: genu	<.0001
		Corticospinal tract right	.0005
		Fronto‐pontine tract right	.0003
		Inferior occipito‐frontal fascicle right	.0006
		Superior thalamic radiation left	.0002
		Superior thalamic radiation right	.0006
		Uncinate fascicle right	.0006
		Thalamo‐premotor right	.0006
		Striato‐fronto‐orbital left	.0005
		Striato‐fronto‐orbital right	.0005
		Striato‐premotor right	.0005
nfvPPA			
Corpus callosum: genu	<.0001	Arcuate fascicle left	<.0001
Cingulum left	.0014	Arcuate fascicle right	.0014
Superior longitudinal fascicle I left	.0008	Anterior thalamic radiation left	.0005
Superior longitudinal fascicle II left	.0006	Anterior thalamic radiation right	<.0001
Thalamo‐premotor left	.0059	Corpus callosum: rostrum	.0001
		Corpus callosum: genu	<.0001
		Corpus callosum: rostral body	.0018
		Corpus callosum: post. midbody	.0007
		Cingulum left	<.0001
		Cingulum right	.0011
		Fronto‐pontine tract left	.0004
		Fronto‐pontine tract right	.0005
		Inferior occipito‐frontal fascicle left	<.0001
		Inferior occipito‐frontal fascicle right	<.0001
		Inferior longitudinal fascicle right	.0011
		Optic radiation left	<.0001
		Optic radiation right	.0001
		Superior longitudinal fascicle I left	<.0001
		Superior longitudinal fascicle I right	.0001
		Superior longitudinal fascicle II left	<.0001
		Superior longitudinal fascicle II right	.0008
		Superior longitudinal fascicle III left	<.0001
		Superior longitudinal fascicle III right	.0012
		Uncinate fascicle left	.0007
		Thalamo‐premotor left	.0002
		Thalamo‐parietal left	.0013
		Thalamo‐parietal right	.0001
		Thalamo‐occipital left	.0008
		Thalamo‐occipital right	.0001
		Striato‐fronto‐orbital left	<.0001
		Striato‐fronto‐orbital right	<.0001
svPPA			
Inferior occipito‐frontal fascicle left	.0018	Arcuate fascicle left	.0023
Superior longitudinal fascicle III left	.0017	Arcuate fascicle right	.0018
		Corpus callosum: rostrum	.0019
		Corpus callosum: genu	.0018
		Corpus callosum: isthmus	.0020
		Cingulum left	.0016
		Inferior occipito‐frontal fascicle left	.0021
		Inferior occipito‐frontal fascicle right	.0016
		Inferior longitudinal fascicle left	.0007
		Inferior longitudinal fascicle right	.0005
		Optic radiation left	.0015
		Optic radiation right	.0018
		Superior longitudinal fascicle I left	.0017
		Superior longitudinal fascicle I right	.0020
		Superior longitudinal fascicle II left	.0015
		Superior longitudinal fascicle II right	.0019
		Superior longitudinal fascicle III left	.0019
		Superior thalamic radiation right	.0019
		Uncinate fascicle left	<.0001
		Thalamo‐parietal left	.0017
		Thalamo‐parietal right	.0018
		Thalamo‐occipital left	.0018
		Thalamo‐occipital right	.0019
		Striato‐fronto‐orbital left	.0019
		Striato‐fronto‐orbital right	.0019

Abbreviations: FA, fractional anisotropy; RD, radial diffusivity.

### 3.3 Validation

**FIGURE 2:**
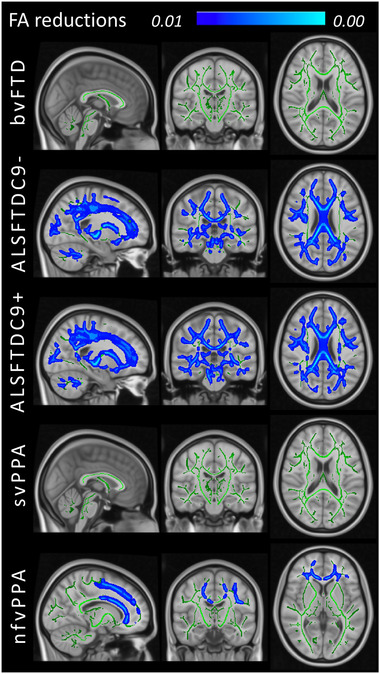
Fractional anisotropy (FA) reductions at group level in patients with behavioral variant FTD (bvFTD), ALS‐FTD patients without *C9orf72* hexanucleotide expansions (ALSFTDC9‐), ALS‐FTD patients with *C9orf72* hexanucleotide expansions (ALSFTDC9+), semantic variant primary progressive aphasia (svPPA) and nonfluent variant primary progressive aphasia (nfvPPA) compared to healthy controls at *p* < .01 controlling for age, sex and family‐wise error

**FIGURE 3:**
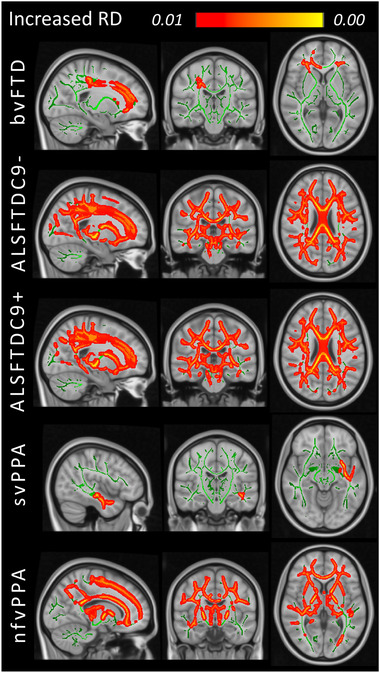
Increased radial diffusivity (RD) at group level in patients with behavioral variant FTD (bvFTD), ALS‐FTD patients without *C9orf72* hexanucleotide expansions (ALSFTDC9‐), ALS‐FTD patients with *C9orf72* hexanucleotide expansions (ALSFTDC9+), semantic variant primary progressive aphasia (svPPA) and nonfluent variant primary progressive aphasia (nfvPPA) compared to healthy controls at *p* < .01 controlling for age, sex and family‐wise error

For validation purposes, standard TBSS analyses were performed to contrast each of the five FTD groups to HCs. Widespread, multilobar FA reductions were detected in both ALS‐FTD groups irrespective of *C9orf72* status (Figure 2). Anterior frontal and left hemisphere predominant FA reductions were identified in the nfvPPA group. At *p* < .01, no significant FA reductions were identified in the svPPA and bvFTD groups. At the same statistical threshold, areas of increased RD were detected in each FTD group: orbitofrontal and forceps minor predominant changes in bvFTD, left superior temporal and insular WM alterations in svPPA and extensive multilobar WM degeneration in nfvPPA, ALS‐FTD‐C9+ and ALS‐FTD‐C9‐. (Figure 3). While RD was more sensitive in detecting WM pathology in bvFTD and svPPA, FA was more sensitive in detecting cerebellar changes in the two ALS‐FTD groups compared to RD. Occipital involvement was relatively limited in the nfvPPA group.

## 4 DISCUSSION

We have successfully captured phenotype‐specific WM alterations in individual FTD patients using a *z*‐scored‐based strategy. The group‐level findings inferred from the trialed WM rating scheme were consistent with the outputs of established pipelines. Our results indicate that it is feasible to interpret single DTI datasets if large reference datasets are available with uniform scanning parameters.

In a clinical setting, gray matter atrophy can often be qualitatively appreciated in patients with FTD (Hardiman et al., 2016). In contrast, WM changes cannot be meaningfully commented upon beyond the exclusion of demyelination, inflammatory or vascular changes. WM changes are typically reviewed visually on T2w, FLAIR and DWI to make sure the suspected diagnosis is not confounded by coexisting vascular, inflammatory or neoplastic, or paraneoplastic pathology. In established cases of FTD, FLAIR and T2w images often look relatively normal, and WM ‘‘atrophy’’ cannot be ascertained on visual inspection. While most FTD phenotypes are associated with the selective degeneration of specific WM tracts, these patterns are not visible on standard clinical sequences. It is also noteworthy that clinical pulse‐sequences are typically optimized for speed of acquisition, often include slice gaps and operate with large voxel sizes, especially for FLAIR and T2w.

In a research setting, imaging traits are typically derived from contrasting a group of patients with a specific clinical profile or a specific mutation to a group of demographically matched controls. In bvFTD, progressive WM changes have been described in the uncinate fasciculus, cingulum and corpus callosum; and to a lesser extent in the anterior thalamic radiation, fornix and superior and inferior longitudinal fasciculus in both hemispheres (Agosta et al., 2012; Borroni et al., 2007; Lam et al., 2014; Mahoney et al., 2014; Whitwell et al., 2010). Studies in nvfPPA captured preferential left‐sided changes in the anterior thalamic radiation, uncinate and superior longitudinal fasciculus (Agosta et al., 2012; Schwindt et al., 2013; Whitwell et al., 2010), which become more prominent in the right hemisphere over time (Lam et al., 2014). In svPPA, left‐hemispheric uncinate, arcuate and inferior longitudinal fasciculus (Agosta et al., 2012; Borroni et al., 2007; Schwindt et al., 2013; Whitwell et al., 2010) degeneration has been consistently detected which remain relatively focal on longitudinal follow‐up with some interval involvement of right frontotemporal regions (Agosta et al., 2012; Lam et al., 2014). In MAPT mutation carriers, early parahippocampal, cingulate and uncus involvement can be detected (Jiskoot et al., 2018, 2019; Rohrer et al., 2010) accompanied by corpus callosum, inferior and superior longitudinal fasciculus and fornix degeneration (Jiskoot et al., 2018; Mahoney et al., 2014; Rohrer et al., 2010). In association with GRN, early corpus callosum and internal capsule changes have been described, followed by left‐hemisphere predominant cingulum, inferior fronto‐occipital, superior and inferior longitudinal fasciculus degeneration (Jiskoot et al., 2018, 2019; Rohrer et al., 2010). *C9orf72* repeats have been linked to corticospinal tract, corpus callosum, thalamic radiation, cingulum, uncinate, superior and inferior longitudinal fasciculus degeneration (Jiskoot et al., 2018; Mahoney et al., 2014). The imaging signatures of rare genotypes—such as *TARDBP* and *VCP*—are poorly characterized as these have been predominantly evaluated in smaller case series (Häkkinen et al., 2020).

The group comparisons of academic imaging have relatively little to offer when the priority is the appraisal of cerebral pathology in individual patients; either in those with a suspected diagnosis or on follow‐up of patients with an established diagnosis. While machine learning (ML) applications show promise in accurate patient categorization, they work best when ample training data are available which pertains to more common neurodegenerative conditions (Grollemund et al., 2020a, 2020b). An advantage of the presented method is that, contrary to ML applications, it does not impose a possible diagnostic label (category), but merely lists the tracts which are ‘‘affected’’ compared to normative controls. This leaves the interpretation of the output text file to the clinicians to be judiciously integrated with clinical findings and the broader clinical context, family history, comorbid conditions, genetic susceptibility and so forth. As shown in Figure 1, the algorithm offers a list of affected tracts based on a single DWI scan which can be depicted visually if needed, but the main output is the text file with the relevant *z*‐scores and *p*‐values.

Another advantage of the approach is the detection of WM abnormalities in each hemisphere separately. The laterality of findings can then be interpreted in single subjects based on handedness which is particularly important in language variant FTDs. Our findings indicate left hemispheric dominant WM pathology in svPPA and nfvPA both at individual and group level. This is in striking contrast to the relatively symmetric WM degeneration observed in ALS‐FTD. Academic studies using group comparisons typically pool data across right‐ and left‐handed subjects which makes the interpretation of the laterality of findings more challenging. The quantitative evaluation of single subjects has other advantages. Pooled group‐level data not only introduce undue heterogeneity in terms of handedness but also with regard to symptom duration and disease severity which undermines the value of group‐level inferences and rendering them less pertinent to single participants. This study has exclusively focused on whiter matter alterations. The assessment of cortical gray matter changes has been previously tested in a similar framework (Tahedl, Chipika, et al., 2021; Tahedl, Li Hi Shing, et al., 2021). It is conceivable that additional imaging measures, such as basal ganglia volumes normalized for total intracranial volume (TIV), alternative WM metrics, metabolite ratios and network coherence indices could be interpreted in a similar framework with reference to normative data (Abidi et al., 2020, 2021; Dukic et al., 2019; Feron et al., 2018; Nasseroleslami et al., 2019; Proudfoot et al., 2018) as well as cord parameters in ALS‐FTD cohorts (Bede et al., 2012; El Mendili et al., 2019; Lebouteux et al., 2014; Querin et al., 2019; Querin et al., 2018). Finally, it is plausible that statistical outputs from imaging modalities can be integrated into larger biomarker panels, which would include quantitative serum, cerebrospinal fluid, electroencephalographic, magnetoencephalographic, proteomic and neuropsychological indices (Blasco et al., 2018; Burke, Elamin, et al., 2016; Burke, Pinto‐Grau, et al., 2016; Christidi et al., 2019; Devos et al., 2019; Dukic et al., 2019; Nasseroleslami et al., 2019; Proudfoot et al., 2018). While the group‐level outputs of the *z*‐scored‐based strategy and TBSS are anatomically concordant, their sensitivity in detecting WM changes is different. It is noteworthy that FA on TBSS does not capture WM degeneration in svPPA and bvFTD even at *p* < .01 using the appropriate covariates. Using the tract‐wise approach, FA reductions are readily detected in the anterior corpus callosum in bvFTD and in the left inferior occipito‐frontal and left superior longitudinal fascicles in svFTD (Table 3). At an individual level, the *z*‐score‐based approach readily detects the degeneration of relevant WM tracts in these two groups, which may be ‘‘averaged out’’ by less severe cases in the group comparisons (Figure 1). TBSS generates voxelwise statistical maps projected on a WM skeleton which can be thresholded at a specific *p*‐value, but it is typically reviewed visually, that is, anything below that threshold is highlighted as ‘‘affected’’ with a color spectrum map. In contrast, the text outputs from the *z*‐score approach offer a list of ‘‘affected tracts’’ which can be ranked in order of ‘‘severity’’ based on associated *p*‐values.

Both the tractwise analyses and TBSS suggest that RD is more sensitive to detect WM alterations in FTD. Based on RD profiles, affected tracts in bvFTD include corpus callosum, corticospinal tract and a number of subcoritco‐cortical projections such as the superior thalamic radiation, thalamo‐premotor and striato‐premotor fibers. The involvement of the corticospinal tract in bvFTD is of interest as another shared feature between ALS and FTD. The involvement of bundles linking subcortical and cortical regions supports previous findings (Chipika, Finegan, et al., 2020) and highlights the contribution of subcortical pathology to clinical manifestations (Christidi et al., 2018). WM degeneration in svPPA not only includes the corpus callosum, cingulum and arcuate degeneration but the left‐hemisphere predominant involvement of long association fibers and projections from the thalamus and striatum (Table 3). The nfvPPA cohort exhibits widespread degeneration of both commissural and long association fibers with slight left hemispheric predominance in addition to thalamic and striatal projections. The *C9orf72* negative ALS‐FTD cohort not only exhibits widespread WM pathology in core ALS‐associated regions such as the corticospinal tracts and corpus callosum, but in line with more recent studies, in the cerebellar peduncles, long association fibers, arcuate fasciculus, uncinate and cingulum (Bede, Chipika, et al., 2021; McKenna, Chipika, et al., 2021; Trojsi, Di Nardo, Caiazzo, et al., 2020) (Table 2). WM degeneration in ALS‐FTD patients carrying the GGGGCC hexanucleotide expansion is comparable to the anatomical patterns observed in *C9orf72*‐negative patients, but is more readily detected by FA reductions (Table 2). These observations highlight that contrary to previous suggestions, severe frontotemporal degeneration and subcortical involvement in ALS are not unique to the C9orf72 genotype.

In the absence of accompanying post mortem and CSF data, the participants of this study were merely categorized clinically. FTD phenotypes arise from different underlying proteinopathies (Chare et al., 2014; Josephs et al., 2011; Snowden et al., 2007); ALS‐FTD is primarily linked to pTDP‐43 (Geser et al., 2011), svPPA is nearly always associated with underlying TDP‐43‐C pathological aggregates (Bocchetta et al., 2020), nfvPPA is commonly associated with 4R tau (Spinelli et al., 2017) and molecular findings in bvFTD are thought to be heterogeneous (Perry et al., 2017). There are a number of study limitations we need to acknowledge, chief of which is the limited normative data at our disposal. Larger reference datasets stratified for narrow age brackets would permit more precise data interpretation. In this pilot study, we have only evaluated two diffusivity indices, but other diffusivity metrics, such as AD (Omer et al., 2017) or non‐Gaussian diffusivity measures (Broad et al., 2019), could also be incorporated in *z*‐score models. Finally, this is merely a cross‐sectional study to test a quantitative, single‐subject data interpretation framework. The natural expansion of this study would be tracking single subjects longitudinally to test whether our approach captures expanding WM pathology in single subjects over time. Notwithstanding these limitations, our findings indicate that our strategy offers valuable clinical insights in single‐subjects and may be potentially developed into a viable clinical and pharmaceutical trial applications.

## 5 CONCLUSIONS

Frontotemporal dementia is associated with subtype‐specific WM signatures, and regional WM degeneration is a key contributor to phenotype‐defining clinical manifestations. The early diagnosis of FTD soon after symptom onset is challenging, and the current clinical role of imaging is limited to the exclusion of alternative structural, inflammatory or neoplastic pathologies. As demonstrated, carefully designed computational pipelines enable the interpretation of individual diffusion datasets and the ascertainment of anatomical patterns of WM degeneration in vivo. The development, optimization and validation of similar imaging frameworks that categorize individual patients based on raw MR data should be a key research priority. These initiatives signal a departure from describing group‐level signatures, and herald a paradigm shift to precision, individualized, computational radiology.

## CONFLICT OF INTEREST

The authors declare no conflict interest.

## AUTHOR CONTRIBUTIONS

*Conceptualization of the study*: Mary Clare McKenna, Marlene Tahedl and Peter Bede. Drafting of the manuscript: Mary Clare McKenna, Marlene Tahedl and Peter Bede. *Neuroimaging analyses*: Mary Clare McKenna, Marlene Tahedl, Aizuri Murad, Jasmin Lope and Peter Bede. *Revision of the manuscript for intellectual content*: Mary Clare McKenna, Marlene Tahedl, AM, JL, OH, SH and Peter Bede.

## Data Availability

The data are not publicly available due to privacy or ethical restrictions.
